# High LET-Like Radiation Tracks at the Distal Side of Accelerated Proton Bragg Peak

**DOI:** 10.3389/fonc.2021.690042

**Published:** 2021-06-10

**Authors:** Dakota Horendeck, Kade D. Walsh, Hirokazu Hirakawa, Akira Fujimori, Hisashi Kitamura, Takamitsu A. Kato

**Affiliations:** ^1^ Department of Environmental & Radiological Health Sciences, Colorado State University, Fort Collins, CO, United States; ^2^ National Institute of Radiological Sciences, National Institutes for Quantum and Radiological Science and Technology, Chiba, Japan; ^3^ Radiation Emergency Medical Assistance Team, National Institutes for Quantum and Radiological Science and Technology, Chiba, Japan

**Keywords:** DNA damage, proton radiotherapy, linear energy transfer, Bragg peak, gamma-H2AX

## Abstract

Proton therapy is a type of hadron radiotherapy used for treating solid tumors. Unlike heavy charged elements, proton radiation is considered to be low LET (Linear Energy Transfer) radiation, like X-rays. However, the clinical SOBP (Spread Out Bragg Peak) proton radiation is considered to be higher in relative biological effectiveness (RBE) than both X-ray and their own entrance region. The RBE is estimated to be 1.1–1.2, which can be attributed to the higher LET at the SOBP region than at the entrance region. In order to clarify the nature of higher LET near the Bragg peak of proton radiation and its potential cytotoxic effects, we utilized a horizontal irradiation system with CHO cells. Additionally, we examined DNA repair mutants, analyzed cytotoxicity with colony formation, and assessed DNA damage and its repair with *γ*-H2AX foci assay in a high-resolution microscopic scale analysis along with the Bragg peak. Besides confirming that the most cytotoxic effects occurred at the Bragg peak, extended cytotoxicity was observed a few millimeters after the Bragg peak. *γ*-H2AX foci numbers reached a maximum at the Bragg peak and reduced dramatically after the Bragg peak. However, in the post-Bragg peak region, particle track-like structures were sporadically observed. This region contains foci that are more difficult to repair. The peak and post-Bragg peak regions contain rare high LET-like radiation tracks and can cause cellular lethality. This may have caused unwanted side effects and complexities of outputs for the proton therapy treatment.

## Introduction

Proton therapy (PT) is a type of hadron radiotherapy for treating mainly solid tumors (1). Accelerated protons have a unique dose distribution along their path due to the nature of hadron radiation. The initial radiation dose is small at the entrance region. However, when protons reach the end of their path, all of the energy is deposited in a region known as the Bragg peak (2). In the post-Bragg peak region, a small amount of dose is produced by the reaction products (2). Therefore, protons can target tumors located in the body without harming the surrounding normal tissues. In general, hadron radiation has a superior dose distribution than conventional photon radiation therapy (3). Among hadron radiation, proton radiation has less of a tail region than carbon-ion radiotherapy and less uncertainty for side effects due to the higher biological effectiveness of carbon ion radiotherapy (4). Therefore, PT is the preferred modality for patients with younger ages to avoid potential secondary tumors (5, 6). However, the proton beam can contains neutron contamination and scattered particles, leading to poorer beam profile (7). Unexpected side effects were recently reported after PT, such as brain injury (6, 8–11).

The proton beam has less tail regions than carbon-ions (12, 13), but utilizing a computer simulation by Monte Carlo calculation suggested some dose distribution after the Bragg peak (14, 15). These tail regions in the proton beam contain relatively high LET particles in a range up to 10 keV/μm, but up to 30 keV/μm (16) or 40 keV/um (17) were also reported. The LET range around 30–40 keV/μm is still not considered as high as the biological maximum LET value of 100 keV/μm, but it can cause a significant increase of relative biological effectiveness (RBE). In our previous studies, carbon-ion monoenergetic beams with LET values between 13 and 30 keV/μm could produce RBE values of 1.1–1.5 (18, 19). Besides RBE, other important cellular responses such as the oxygen enhancement ratio (OER) can also be slightly affected by radiation within this range of LET (18). LET values in the proton entrance region are approximately 1 keV/μm and cannot result in high RBE or low OER (20). Currently, the RBE of clinical proton beams in the proton SOBP region is estimated to be approximately 1.1 to 1.2 (7, 21–23).

In order to clarify the true nature of the proton RBE from biological responses at the Bragg peak and the surrounding area, a position dependent analysis was carried out with 0.5 mm to a few millimeter increments to cover the proton beam paths (24–27). We utilized a horizontal irradiation system, which we previously developed (28). This irradiation system can visually show cellular cytotoxic locations in the flasks. Additionally, we combined it with a microscopic analysis to clarify DNA damage and distribution near the Bragg peak to detect any specific changes in this narrow area. Interestingly, DNA damage with track structures produced by protons and fragments can be a good indicator of energy deposition/LET of the fragments (29). Without using expensive deconvolution software or super high-resolution microscopy, clustered foci can be denoted as a particle track-like structures by using this method (30, 31). Monoenergetic proton beams in this study will provide clear dose and LET distribution along their path. The findings in this study will provide micro-bio-dosimetry analysis for the biological significance of the proton beam.

## Materials and Methods

### Cell Culture

CHO wild type (CHO 10B2) was kindly supplied by Dr. Joel Bedford of Colorado State University (Fort Collins, CO, USA). DNA repair deficient CHO mutants, V3 (DNA-PKcs, non-homologous end joining repair deficient) (32) and 51D1 (Rad51D, homologous recombination repair deficient) (33) were kindly supplied by Dr. Larry Thompson at the Lawrence Livermore National Laboratory (Livermore, CA, USA). Cells were maintained in Alpha-MEM (ThermoFisher, Waltham, MA) with 10% heat inactivated Fetal Bovine Serum (Sigma, St. Louis, MO), antibiotics (Anti-Anti; Invitrogen, Grand Island, NY) and were cultured in 37°C incubators with 5% CO_2_ and humidity. We utilized CHO cells rather than human cells for the following reasons (1): colony size and shape: CHO cells produce dense, tightly packed colonies and the colony shape of CHO cells is very circular. On the other hand, colonies of many cells of human origin often spread flat and large and form uneven shapes. In this manuscript, the location of survival colonies has to be accurately recorded. Therefore, using CHO cells was of the utmost importance.

### Irradiation

Proton beam irradiation was conducted at the QST (National Institutes for the Quantum and Radiological Sciences and Technology) in Chiba, Japan. Protons were accelerated to 70 MeV using the NIRS-930 cyclotron (24). Proton beam was delivered for the circular field of 7 cm diameter with 95% uniformity. Dose rate was set at 3 Gy/min. Monoenergetic 70 MeV protons have a LET value of 1 keV/μm on entrance. Exponentially growing cells were irradiated at room temperature. Dosimetry was carried out with a Markus ion chamber (PTW 23343, PTW, Freiburg GmbH, Germany) with the container filled with water or complete cell culture media. The LET values were calculated by SRIM (Stopping and Range of Ions in Matter) program from the range of the proton beam (16).

**Figure 1:**
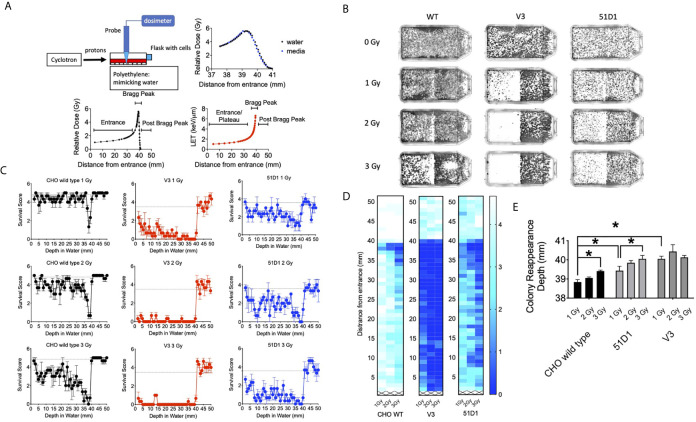
Colony formation after horizontal proton irradiation. **(A)** Proton irradiation set-up and dose distribution measurement and calculated LET values of protons from the entrance, Bragg peak, and post-Bragg peak. The black lines indicate relative doses in water; the blue points indicate relative doses in cell culture media, and the red line indicates the calculated LET values. **(B)** Representative images of colony distribution after 0–3 Gy of initial proton irradiation to CHO wild type, V3, and 51D1 cells. The proton beam traveled from left to right. **(C)** Cell survival score after 0–3 Gy of initial proton irradiation to CHO wild type, V3, and 51D1 cells. Dashed lines represent the unirradiated control. **(D)** Heat map of cytotoxicity after proton radiation. Maximum cytotoxicity was observed at 38 mm with 35–41 mm from the entrance. The first 1 mm represents the flask wall. The right bar, scaled 0–4, indicates that a cell survival of 0 represents cell death, while 4 indicates the highest cell survival. **(E)** Colony reappearance range in different radiosensitive cells. Error bars indicate the standard error of the means. * means statistically significant differences (P < 0.05).

Irradiation was carried out as previously described (28) (**Figure 1**). Prior to irradiation, cell culture flasks or SlideFlasks were placed upright with the capped end opposite to the proton beam source. The thickness of the flask and SlideFlask was 1 mm of polystyrene, which is equivalent to water thickness of 1.0368 mm (34). Therefore, the analysis started 1 mm from the proton entrance for cell survival analysis. The geometric location of the SlideFlask was matched with a micrometer and an M Processor (LASICO, Los Angeles, CA) geometric recorder.

### Colony Formation and Manual Colony Distribution Analysis

Two hours before irradiation, 10,000 cells were plated onto a T25 flask, which has 25 cm^2^ of growing area to produce a density of an average of four cells per mm^2^. After irradiation, cells were disturbed minimally during transportation from the irradiator to the incubator and kept in an incubator for 8 days to form colonies. Colonies were fixed and stained 8 days later using 100% ethanol followed by 0.1% crystal violet. Macroscopic colonies containing more than 50 cells were marked as survivors (35). The cellular attachment was confirmed after testing medium changes at different times. No colonies were observed at the highest dose Bragg peak region, which supports that there were no-floating cells during the trip from irradiation to incubation.

For a rough geometrical analysis of colony distribution, locations of survivors were recorded with a ruler. The flasks used have a wall that is 1 mm thick. From the end of the flask, the proton beam entry side for every 1 mm of colony existence was judged and recorded from the entrance up to 50 mm. Five lines were analyzed per flask. The survival score was defined as the presence of colonies at each distance. Five evenly different locations were analyzed with a ruler for the presence or absence of colonies. The survival score of five indicated the representation of all of the colonies that survived. The colony distribution was presented in graphs and in a heat map with Graphpad Prism 8 software (GraphPad, La Jolla, CA, USA).

In order to evaluate the cytotoxic range of the proton beam and maintain a fine geometrical analysis of the colony distribution, the reappearance of colony formation following the Bragg peak was recorded with a ruler. Colony reappearance was defined as the average distance from the entrance for the first observable colonies after the Bragg peak. Thirteen lines were analyzed for each flask to obtain a sensitive analysis of the extension of the cytotoxic range.

### Digital Colony Distribution Analysis With MATLAB

To eliminate the risk of subjective analysis of manual counting, three-dimensional surface plots were created using MATLAB software. Flasks were imaged with the BIO-RAD ChemiDoc chemiluminescent imager (BIO-RAD, Hercules, CA) *via* ImageLab 2.0.1 software (BIO-RAD, Hercules, CA) under epi-white, trans-white illumination utilizing a copper stain emission filter. These images were visualized using intense bands and converted into black and white .JPG formats. The files were cropped to exclude ridges of the T-25 flasks and narrowing neck of the bottle. These images were entered into an executable script created previously (28) *via* the MATLAB software (MATHWORKS, Natick, MA). The script allows .JPG files to be analyzed by pixel shade to create three-dimensional surface plots that can be adjusted to create virtual cell survival plots.

### DNA Damage Distribution Analysis

In order to estimate proton irradiation induced DNA damage and repair, *γ*-H2AX foci were used for a DNA double strand break marker (36–38). CHO wild type cells were plated on a SlideFlask (ThermoFisher) the day before irradiation. This did not change the cell cycle distribution compared to re-plating 2 h before irradiation. At 30 min and 24 h after irradiation, cells were fixed in 4% paraformaldehyde for 15 min, washed three times in PBS for 10 min each, permeabilized for 5 min in 0.2% Triton X-100, and blocked with 10% goat serum in PBS overnight at 4°C. The cells were incubated with anti-*γ*-H2AX mouse monoclonal antibody (Upstate, Charlottesville, VA) for 1 h, washed three times in PBS for 10 min each, and incubated with Alexa Fluor-conjugated goat anti-mouse secondary antibody (Molecular Probes, Eugene, OR) for 1 h at 37°C. Cells were washed four times in PBS for 10 min each and mounted by using DAPI in Prolong Gold (Molecular Probes). Multi-dimensional fluorescence images were captured by using a Zeiss Axioplan fluorescent microscope (Zeiss, Jena, Germany) with a motorized z-stage and CoolSNAP HQ Cooled CCD camera (Photometrics, Tucson, AZ) and Metamorph software (Molecular Devices, San Jose, CA). The microscope was equipped with an M Processor (LASICO, Los Angeles, CA) to record the geometric location of slides.

Images were captured every 3.69 mm from the entrance of the protons to near the Bragg peak and every 0.46 mm or 0.92 mm from the Bragg peak to the post-Bragg peak. At each data point, the number of *γ*-H2AX per cell was manually obtained from at least 30 cells per experiment for the quantitative analysis. In order to investigate the repair-ability of foci at the different depths, the residual foci number was divided by the initial foci number. A track-like structure of DNA damage distribution was visually observed as a solid or dashed line of foci, which was also obtained quantitatively, per cell to estimate intermediate-high LET radiation induced damage.

### Statistical Analysis

Experiments were conducted independently three times. The survival score was obtained from five locations. The colony reappearance was obtained from 13 locations, and at least 30 cells were analyzed for foci analysis. All experimental data was analyzed *via* Prism 8 software. One-way analysis of variance (ANOVA) and Dunnett’s multiple comparison test were conducted for statistical significance. P-values of <0.05 were considered to indicate differences that were statistically significant.

## Results

### Extension of Cytotoxicity Beyond Bragg Peak of Proton Beam

The 70 MeV proton beam has approximately 39 mm of range in water (**Figure 1A**). At 39.4 mm, the relative dose reached 4.12 Gy, and the mean LET values were calculated by SRIM software as 6.59 keV/μm (**Figure 1A**). The horizontal irradiation system visually presented the cell death at the Bragg peak of the proton beam as a gap devoid of colonies with the colony formation assay (**Figure 1B**). The cell survival score test and heat map analysis presented that CHO wild type had maximum cytotoxicity between 37 and 39 mm, where the lowest survival scores were found (**Figures 1C, D** **)**. At 3 Gy of initial irradiation, elevated cytotoxicity was observed from 34 to 39 mm. There are no clear signs of cellular cytotoxicity after 41 mm for the CHO wild type. Radiosensitive DNA repair deficient mutants V3 and 51D1 showed an even greater reduction of surviving colonies. Overall, they denoted the extension of the cytotoxic range. At 40 mm, the survival scores decreased a statistically significant amount compared to the un-irradiated control (P < 0.01).

Additionally, the extension of the cytotoxic range was analyzed more precisely based on the reappearance of colonies after the Bragg peak (**Figure 1E**). CHO wild type showed the reappearance of colonies at 38.5 mm for 1 Gy and 39.5 mm for 3 Gy. Statistically significant extension was observed between them (p < 0.05). 51D1 also showed reappearance of colonies at 3.93 mm for 1 Gy and 40 mm for 3 Gy, and increased doses extended the cytotoxic range with statistical significance (p < 0.05). Additionally, the location of reappearance for 51D1 cells was extended compared to the CHO wild type (p < 0.05). V3 showed reappearance of colonies at 40 mm for 1 Gy with statistically significant extension compared to the CHO wild type, but 3 Gy of initial irradiation did not extend the reappearance of colony location. This geometric recording of the survival analysis data showed that proton induced cellular lethality was produced beyond the Bragg peak. The additional lethality was observed in the 39 to 40.5 mm region. Since double strand break repair deficient mutants showed additional cytotoxicity compared to repair proficient wild type cells, involvement of DNA double strand break formation is suggested. Since V3 did not show any additional cytotoxicity after 2Gy, it may suggest that the “dose” of fragments causing DNA damage are rapidly decreased after the end of the Bragg peak.

**Figure 2:**
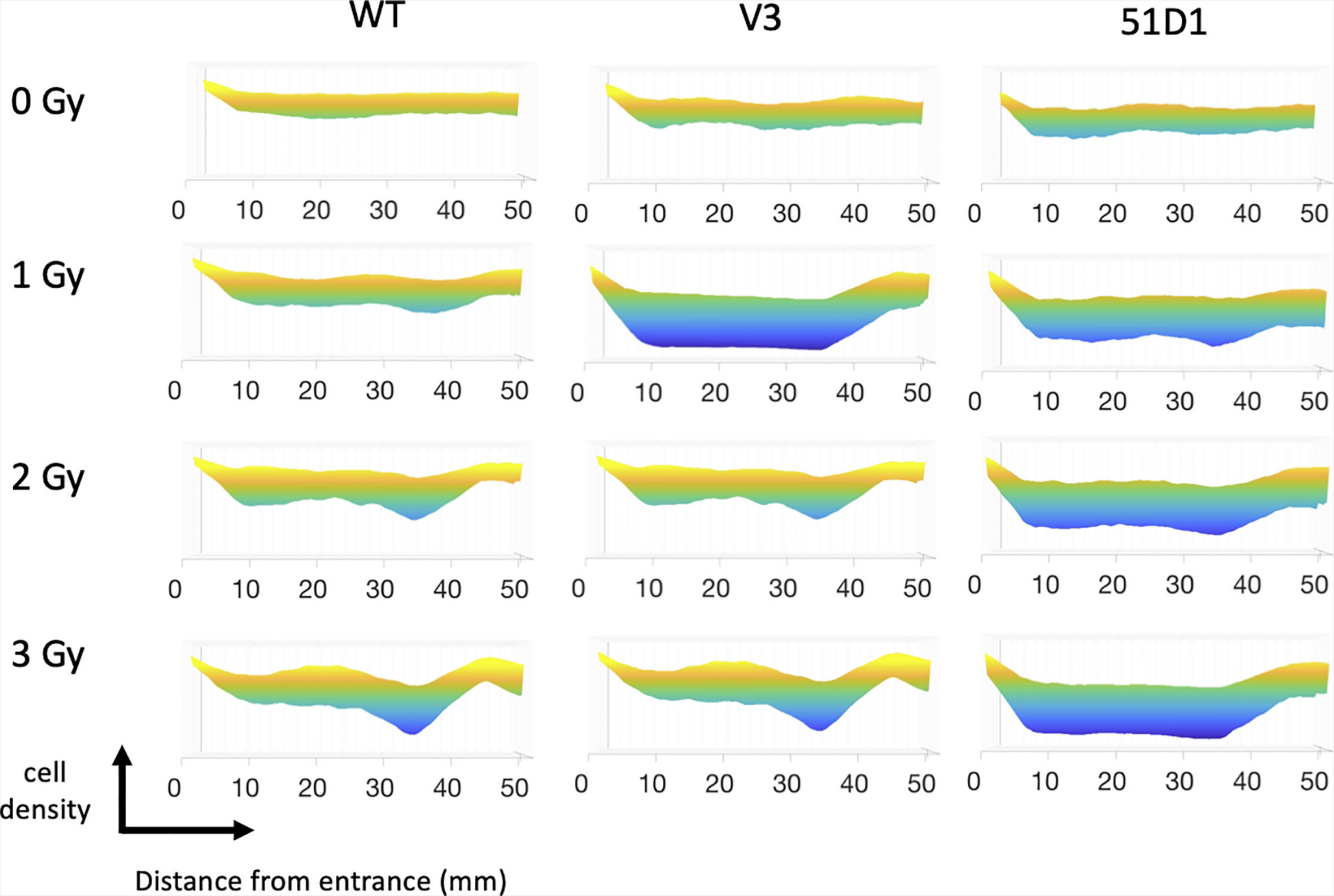
MATLAB image analysis of cell survival after proton irradiation. Yellow color indicates more cells; blue color indicates less cells. Three flasks were merged for analysis.

The survival analysis was confirmed with a digital image analysis to avoid any subjective colony counting (**Figure 2**). This analysis is based on the survived cellular density, not the clonogenic activity measured by colony formation as manual scores. Ultimately, while survivor colonies provide cellular density, both analyses should be very close together. The most cell deaths were observed at the site on the monoenergetic Bragg peak, at 38 mm. Survival plots show a strong correlation with the data obtained from survival score graphs. This shows the ability of the MATLAB software to make an effective analytical tool for rapid analysis. The biggest difference between manual analysis and computer analysis is the tail region. After the Bragg peak, manual counting showed a complete return to background level of clonogenic ability at 40 mm even for radiosensitive cells. On the other hand, the recovery of the computer analysis of the density of cells was slower. This implies that colonies are formed, but may be smaller in size due to the small amount of non-lethal DNA damage and additional support for fragment induced damage after the Bragg peak.

### *γ*-H2AX Foci Distribution Beyond Bragg Peak of Proton Beam

**Figure 3:**
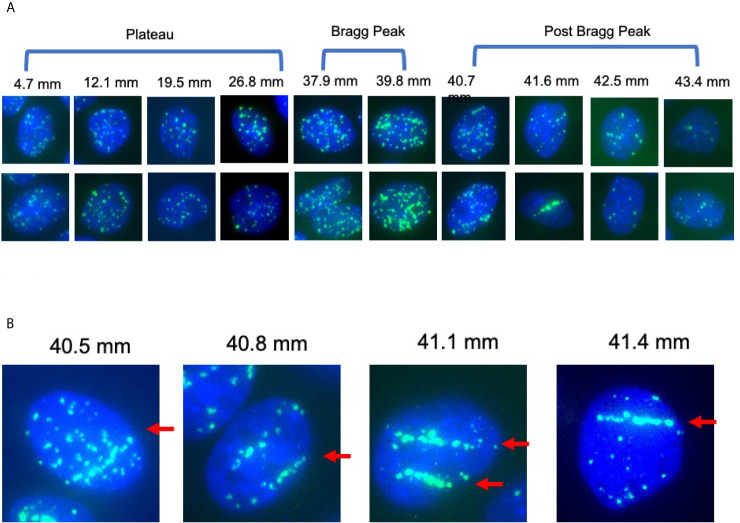
γ-H2AX foci after 1 Gy of proton beam irradiation for CHO wild type cells. **(A)** Representative images of foci number and patterns at the specific distance for CHO wild type cells after proton 1 Gy irradiation. Two images were chosen for each distance. **(B)** Representative images of foci alignment like a track structure for CHO wild type cells after 1 Gy of proton beam. Green signals indicate *γ*-H2AX foci. Blue signals are nuclei stained with DAPI. Arrows indicate track like structures of foci distribution.

DNA damage, especially in the form of DNA double strand break, is the most reasonable way to cause cytotoxicity beyond the Bragg peak of the proton beam. The fragments of targets including proton, neutron and electrons can cause ionization and DNA breaks (2). Therefore, DNA damage was quantitatively or qualitatively analyzed, with number and distribution of *γ*-H2AX foci at the specific location corresponding to the proton beam path (**Figure 3A**). Track-like line alignments were sporadically in 10–30% of cells observed near the Bragg peak, especially between 39 and 42 mm, where clear line-like foci alignment was visible (**Figure 3B**).

**Figure 4:**
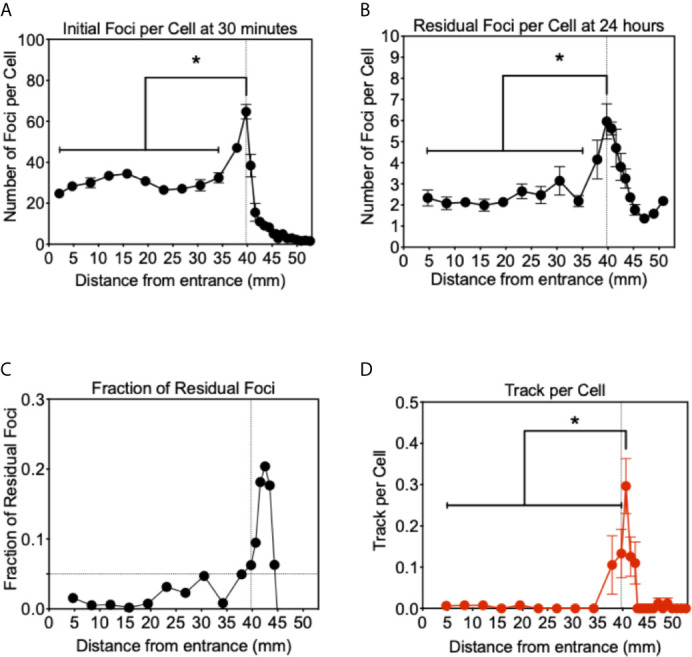
Analysis of *γ*-H2AX foci after proton beam irradiation for CHO wild type cells. **(A)** Initial DNA damage 30 min after 1 Gy of irradiation. **(B)** Residual DNA damage 24 h after 1 Gy of irradiation. **(C)** Fraction of residual DNA damage obtained by residual foci number divided by initial foci number. **(D)** Track like foci pattern formation at 30 min after irradiation. Vertical lines indicate the peak of Bragg peak at 39.75 mm of initial foci formation. Horizontal line indicates the fraction of residual foci of 0.05. Error bars indicate the standard error of the means. * indicates statistically significant differences (P < 0.05).

For the initial DSB formation after 1 Gy, *γ*-H2AX foci formation was analyzed 30 min after irradiation (**Figure 4A**). From the entrance of the proton beam to 34.2 mm, the number of *γ*-H2AX foci was steady at approximately 30 foci per cell. At the Bragg peak region, 49 foci per cell at 37.9 mm and 64 foci per cell at 39.7 mm were observed and increases were statistically significant when compared to the entrance region (P < 0.05). After the Bragg peak, the foci number rapidly decreased and returned close to the background level of 3.5 foci per cell. At 40.7 mm, 36 foci per cell and 15 foci at 41.6 mm were observed. Generally, not many foci were observed after the Bragg peak.

Twenty four hours after irradiation, the number of residual foci was analyzed in the same manner (**Figure 4B**). Foci number was dramatically reduced at all points compared to initial number of damages. From the entrance to 34.2 mm, approximately two foci per cell were observed and no statistically significant increase was observed compared to the foci number of the control. At the Bragg peak region, 39.7 mm, a statistically significant greater number of foci than the entrance were observed as 6.0 foci per cell (P < 0.05). Beyond the Bragg peak, a noticeably greater number of foci were seen compared to the initial foci, which rapidly decreased after the Bragg peak. Track-like foci alignments were not observed in the cells 24 h after irradiation.

The greater number of residual foci may be simply attributed to the higher doses initially irradiated near the Bragg peak. In order to normalize and obtain a fraction of residual foci, we divided the residual foci number by the initial foci number (**Figure 4C**). These un-repaired residual foci are highly associated with complex or clusters of DNA damage that can resemble HZE (high atomic number and energy) particle irradiations. The fraction of residual foci was approximately 0.01 to 0.1 at the entrance region. The fraction of residual foci was increased near the Bragg peak. In particular, a fraction of 0.05 and above was observed from 39.7 to 44.4 mm. It was similar to the Bragg peak of the proton beam observed with initial damage at 39.7 mm, but shifted beyond the Bragg peak. This may be associated with DNA damage produced at the Bragg peak, while the slightly extended post-Bragg peak contained complex DNA damage that is difficult to be repaired.

In order to understand unrepaired residual foci at the post-Bragg peak region, foci distribution was qualitatively analyzed. Sporadically track-like structures of DNA damage were observed in 10–30% of cells near the Bragg peak (**Figure 4D**). The track number per cell was obtained to estimate DNA damage with the track structure, which may be associated with higher LET than regular 1 keV/μm. The track structure was seen exclusively from 37.9 to 42.5 mm with the highest fraction at 40.7 mm. The distribution of the tracks per cell was also shifted to the post-Bragg peak region. This suggests that track DNA damage should contribute to the stronger biological effectiveness of protons. The distribution of unrepaired foci and track was seen up to 42.5 mm. This is matched with the cellular toxicity observed in DNA repair deficient cells (**Figure 1C**).

## Discussion

Proton therapy (PT) is favorable when compared to photon therapy because PT uses the same low LET radiation and focuses dose distribution to tumors more effectively (2). For CIRT, (Carbon Ion Radiotherapy) it may be dangerous to have high LET components with unexpected side effects, including secondary tumors and stronger late effects with a longer tail range and uncertainty of biological effects (3). The present work with horizontal irradiation to a monolayer cell culture showed that the proton beam has minimal effects, but enough to cause cytotoxicity in the post-Bragg peak region (**Figure 1**). As previously shown, our results confirmed that a LET increase occurs at a greater depth slightly beyond the Bragg peak, resulting in a small extension of the biologically effective dose (12). The nature of the post-Bragg peak region of the proton seems interesting. It is obviously much lower in dose than the entrance and the Bragg peak region. However, within a few millimeters after the Bragg peak of a 70 MeV proton beam, it delivers relatively higher LET radiation and damage that effectively cause lethality to cells. Using a clinical proton SOBP beam with stronger energy and longer paths of protons, the post-Bragg peak effect may be observed in a wider area than that currently studied. Horizontal irradiation to the three dimensional target systems such as phantom will provide more information in future. Although the track-like structure of foci produced by proton radiation at the distal edge and the post-Bragg peak region was not as frequently observed as carbon ion or other HZE particles (**Figure 3**), some of them resembled HZE induced dense track-like foci patterns (29), which may explained previously reported higher RBE along distal edge of proton Bragg peak (39, 40). In the clinically relevant doses of irradiation tested in this study, an average of 6.59 keV/μm of LET values was calculated at the Bragg peak (**Figure 1A**), but not all cells had tracks and are relatively rare and sporadic events. This suggests that the cells at the Bragg peak and the post-Bragg peak regions would be irradiated with very heterogenic LET qualities of radiation and might respond differently depending on the damages produced by low to high LET irradiation. It is not a surprise that researchers could not find the significant biological effectiveness in the post-Bragg peak region with the standard colony formation assay, that is unless the dose distribution profile of irradiation was conducted with at least a millimeter sensitivity or horizontal irradiation (24, 25), both of which were successfully achieved in this study. Heterogenic DNA damage amount and distribution were observed by foci analysis near the Bragg peak. These damages cause not only cytotoxicity but also genotoxicity, which may increase normal tissue complication probability. Further analysis needs a low background and high induction assay such as reporter assays to confirm the biological effects other than cytotoxicity at the post-Bragg peak. Moreover, the nature of fragments should be clarified because the proton cannot be disintegrated into smaller fragments as heavy charged particles. The particles causing high LET track-like structures at the post-Bragg peak region may be recoiled neutrons or scattered protons. This needs to be confirmed with advanced physics instruments (41).

With slightly higher LET values, should PT be discouraged? The secondary tumor risk from this middle range LET radiation may answer this (4). However, this finding provides useful information for proton radiotherapy. If the Bragg peak contains a significant fraction of intermediate range LET (10–30 keV/μm) or higher LET as observed for foci patterns, this will answer why RBE values are 1.1–1.2 and slightly higher than the plateau region and photon radiation (7, 21, 22). From the foci patterns, the intermediately high LET portion of the proton beam is limitedly distributed at the narrow region near the Bragg peak. Therefore, the distal portion of the SOBP should be rich in high LET radiation and is expected to have higher RBE as previously shown (39, 42). However, within the SOBP region getting wider, this high LET radiation would be diluted with abundant low LET protons. If treatment can be conducted with multiple short SOBP from multiple directions, proton therapy could gain the advantage over CIRT partially. It will have lower oxygen effects and higher RBE effects. Due to limited LET value, it is hard to expect the same degree of advantage from CIRT. The degree of improvement is still unclear, but it is worth investigating for the future. Additionally, in order to decrease the potential side effects, the distal portion after the SOBP should be monitored with extra caution to determine the irradiation volume.

In conclusion, the horizontal irradiation confirmed that the Bragg peak and slightly shorter range of the post-Bragg peak region of proton radiation contain relatively high LET radiation and induce significant biological effectiveness. This may be due to complex DNA damage produced with a track-like structure observed near the Bragg peak. This finding may explain the partially unwanted side effect observations, but proton therapy can be improved with a narrower SOBP treatment.

## Data Availability Statement

The original contributions presented in the study are included in the article/supplementary material. Further inquiries can be directed to the corresponding author.

## Author Contributions

Conceptualization, TK. Methodology and formal analysis, DH, KW, HK, TK, and AF. Resources data curation, DH, KW, HH, HK, and TK. Writing—original draft preparation, DH and TK. Writing—review and editing, KW and TK. Funding acquisition, AF and TK. All authors contributed to the article and approved the submitted version.

## Funding

This research was partially funded by Dr. Akiko Ueno Radiobiology Fund (TK) and Japan Ministry of Education, Culture, Science and Technology (MEXT) Grants-in-Aid for Scientific Research on Innovative Areas (JP15K21745, AF).

## Conflict of Interest

The authors declare that the research was conducted in the absence of any commercial or financial relationships that could be construed as a potential conflict of interest.
